# Daptomycin exposure precedes infection and/or colonization with daptomycin non-susceptible enterococcus

**DOI:** 10.1186/2047-2994-1-19

**Published:** 2012-05-29

**Authors:** Jeremy C Storm, Daniel J Diekema, Jennifer S Kroeger, Sarah J Johnson, Birgir Johannsson

**Affiliations:** 1 University of Iowa Hospital and Clinics, Iowa City, IA, USA; 2 Carver College of Medicine, University of Iowa, Iowa City, IA, USA; 3 College of Public Health, University of Iowa, Iowa City, IA, USA; 4 College of Pharmacy, University of Iowa, Iowa City, IA, USA; 5 Department of Internal Medicine, Division of Infectious Diseases, SW 54–11, General Hospital, 200 Hawkins Drive, Iowa City, IA, 52242, USA

**Keywords:** Enterococcus, Daptomycin, Resistance, Non-Susceptible, DNSE

## Abstract

**Background:**

Daptomycin non-susceptible enterococci (DNSE) are emerging as an important cause of healthcare-associated infection, however little is known about the epidemiology of DNSE. At the University of Iowa Hospitals and Clinics (UIHC) an increase in the frequency of patients infected and/or colonized with DNSE has occurred. The goals of this study were to evaluate potential factors associated with the development of DNSE colonization and/or infection and to compare the characteristics of patients with prior daptomycin exposure to those without prior daptomycin exposure.

**Methods:**

The study is a retrospective case-series involving all patients with DNSE infection and/or colonization at UIHC, a 734-bed academic referral center, from June 1, 2005 to June 1, 2011.

**Results:**

The majority of patients with DNSE colonization and/or infection had prior daptomycin exposure (15 of 25; 60%), a concomitant gastrointestinal process (19 of 25; 76%), or were immunosuppressed (21 of 25; 84%). DNSE infection was confirmed in 17 of 25 (68%) patients, including 9 patients with bacteremia. Twelve of 17 (71%) patients with DNSE infection had prior daptomycin exposure, including 7 of 9 (78%) patients with bacteremia. Compared to patients without prior daptomycin exposure, patients with prior daptomycin exposure were less likely to harbor *E. faecalis* (0% vs. 33%; p = 0.019). A high proportion of patients (10 of 25; 40%) died during their hospitalizations. Most enterococcal isolates were *E. faecium* (86%), and were vancomycin-resistant (72%). Molecular typing revealed a diverse population of DNSE.

**Conclusions:**

Prior daptomycin exposure, immunosuppression, and/or a concomitant gastrointestinal process, may be associated with the development of DNSE. PFGE revealed a diverse population of DNSE, which along with both increasing numbers of DNSE detected yearly and increasing annual rates of daptomycin usage, suggests the emergence of DNSE under antimicrobial pressure.

## Background

Enterococci are Gram-positive, facultative anaerobes that reside primarily in the gastrointestinal tract. They are generally considered to be of low virulence, but are associated with serious bloodstream, joint, wound, cardiac, urinary, and gastrointestinal infections [1]. Enterococci are often multi-drug resistant, and are responsible for the transmission of various genetic resistance elements to other bacteria, including vancomycin resistance to other enterococci as well as *Staphylococcus aureus*[2,3].

Daptomycin is a lipopeptide antibiotic currently approved by the Food and Drug Administration (FDA) for the treatment of complicated infection of skin and/or subcutaneous tissue as well as bacteremia and right-sided endocarditis due to *S. aureus*[3]. In addition, it is often used in the treatment of infections due to vancomycin-resistant enterococci (VRE), although it is not approved for these conditions [3,4]. Shortly after its FDA approval in 2003, reports of infections due to daptomycin non-susceptible enterococci (DNSE) emerged and cases have been described both with and without prior daptomycin exposure [4-9]. Despite this, little is known about the potential causes or risk factors for the development of DNSE colonization or infection. Here, we report the findings at our institution, The University of Iowa Hospitals and Clinics (UIHC), over a 6-year period (June 1, 2005 through June 1, 2011).

## Methods

The UIHC is a 734-bed, academic, and major referral center for the state of Iowa and surrounding region, with over 28,000 inpatient admissions and 900,000 outpatient visits each year. The UIHC Institutional Review Board granted approval for this study.

By searching our clinical microbiology laboratory database, we identified all DNSE isolates from the time the first clinical isolate was identified (June 1, 2005) to June 1, 2011. An extensive review of the medical record was performed for all patients with DNSE isolated from any source. Infection due to DNSE was defined as isolation from a sterile source, or isolation from a non-sterile source if accompanied by documented symptoms or signs of infection and/or treating clinician explicit diagnosis. Daptomycin usage during the study period was determined yearly by dividing the number of inpatients receiving daptomycin (based on discharge billing data) by the total number of patient discharges for a given year. P-values were calculated with the use of the chi-square.

At UIHC, daptomycin susceptibility testing is performed on all enterococcal isolates (both VSE and VRE) according to standard Clinical and Laboratory Standards Institute (CLSI) method [10], with daptomycin non-susceptibility defined as a daptomycin minimum inhibitory concentration (MIC) > 4 micrograms/mL. All enterococcal isolates not susceptible to daptomycin upon initial testing were re-tested using the CLSI broth microdilution and Etest methods. All available DNSE isolates underwent pulsed-field gel electrophoresis (PFGE) to assess for genetic relatedness, using previously described methods [11]. PFGE patterns were analyzed using Bionumerics software (Applied Maths, Kortrijk, Belgium). The unweighted pair group method with arithmetic averages and DICE coefficient (0.5% optimization, 1.0% position tolerance) were used for dendrogram construction. A similarity coefficient of 0.8 was used to define PFGE types, and subtypes were defined as isolates sharing indistinguishable banding patterns.

## Results

The median age of patients with DNSE was 52.2 years, with 14 of 25 (56%) being female (Table 1). DNSE was identified in 25 patients and found in 32 clinical samples from 4 specimen sources including; blood (9 patients; 12 isolates), urine (12 patients; 14 isolates), peritoneal fluid or intra-abdominal abscess (3 patients; 4 isolates), and wounds (2 patients; 2 isolates). One patient had DNSE identified from both urine and blood (Table 1). Multiple isolates from the same patient were always of the same species, PFGE type or subtype, and had an identical antimicrobial susceptibility pattern.

**Table 1:** Clinical characteristics of patients with DNSE colonization or infection

**Year, Age, Sex**	**Past Medical History**	**Total Days of Daptomycin Exposure**	**Species**	**Source**	**Reason for Admission and/or Complications**
2011					
55F	MDS, SCT	5	E. faecium	Blood, urine	Neutropenia, GVHD of gut
55F	ALL, BMT, DM, PVD	No	E. faecium	Blood	Neutropenia, CDI
58F	Gynecological malignancy	9	E. faecium	Blood	GI inflammation due to metastatic disease
59F	AML	19	E. faecium	Blood	Neutropenia, appendicitis
51F	AML, SCT	29	E. faecium	Urine^a^	Neutropenia, colitis
2010					
74F	MDS, SBO, CHF, Pulmonary Htn	10	E. faecium	Urine^a^	Small bowel resection, sepsis
80F	CAD, COPD, PVD	No	E. faecalis	Urine^a^	Bowel ischemia after surgery
42 M	DM, bipolar disorder	12	E. faecium	Wound, lumbar	Chronic lumbar abscess
56F	AML, SCT, DM	67	E. faecium	Blood	Neutropenia, sigmoid colon perforation
2009					
50F	AML, SCT	20	E. faecium	Urine^a^	Neutropenia, GVHD of gut, colitis
50 M	DM, Hip SSTI/osteomyelitis	19	E. faecium	Wound, hip	Hip SSTI / osteomyelitis
49 M	CAD, CHF, COPD, ESRD/HD, PVD	52	Not able to identify	Peritoneal fluid	GI perforation, peritonitis
37 M	Kidney-pancreas transplant, DM	11	E. faecium	Blood	Recurrent cholangitis
49 M	Kidney-pancreas transplant, DM	12	E. faecium	Blood	Intraabdominal abscess after surgery
2008					
39 M	Hydrocephalus, VPS	25	E. faecium	Peritoneal fluid	GI perforation following MVA
61F	DM, endometrial malignancy	No	E. faecium	Urine	Pelvic exenteration, kidney abscess
78 M	CAD, CKD, CVA, DM	No	E. faecium	Urine^a^	Septic arthritis due viridans streptococci
57 M	Urinary bladder malignancy	No	E. faecium	Peritoneal fluid	Pelvic abscess after surgery
2007					
59 M	MDS, BMT	8	E. faecium	Blood	Neutropenic fever, GVHD of gut
59F	DM, ESRD/HD, VHD	16	E. faecium	Urine^a^	Cryptococcal meningitis
2006					
6F	Recurrent UTI	No	Not Available	Urine	UTI
46F	Cholangitis, DM, ESRD/HD	No	E. faecalis	Blood	Recurrent polymicrobial cholangitis
62 M	CAD, COPD	No	Not available	Urine^a^	Lumbar pain and sciatica
2005					
38 M	DM, metastatic malignancy of colon	No	Not available	Urine	Chemotherapy, neutropenia, pyelonephritis
35F	DM, gastroparesis	No	Not available	Urine^a^	Recurrent gastroparesis

Patient characteristics, past medical history, daptomycin exposure history (total days in prior year), isolate species, isolate source, and abbreviated reason for admission and/or complications arising during index admission for the 25 patients identified to be colonized or infected with DNSE during the study period.

NOTE:

a – Represents DNSE colonization.

Abbreviations: ALL = acute lymphoblastic leukemia; AML = acute myelogenous leukemia; BMT = bone marrow transplant; CAD = coronary artery disease; CDI = *Clostridium difficile* infection; CHF = congestive heart failure; CKD = chronic kidney disease; COPD = chronic obstructive pulmonary disease; CVA = cerebro-vascular accident; DM = diabetes mellitus; ESRD/HD = end stage renal disease requiring hemodialysis; GI = gastrointestinal; GVHD = graft versus host disease; MDS = myelodysplastic syndrome; MVA = motor vehicle accident; PVD = peripheral vascular disease; SBO = small bowl obstruction; SCT = stem cell transplant; SSTI = skin and soft tissue infection; UTI = urinary tract infection; VHD = valvular heart disease; VPS = ventriculoperitoneal shunt; Pulmonary Htn = pulmonary hypertension.

Infection due to DNSE was confirmed by both laboratory and chart review in 17 of 25 (68%) patients. In the remaining 8 patients, DNSE was isolated from urine alone and represented colonization based on the absence of documented clinical symptoms and/or negative urinalysis. A bloodstream infection was identified in 9 patients, a genitourinary infection such as UTI, pyelonephritis, or kidney abscess in 3 patients, bacterial peritonitis in 3 patients, and a skin and soft tissue infection and/or osteomyelitis in 2 patients.

A concomitant gastrointestinal or intra-abdominal process was identified in 19 of 25 (76%) patients, including *Clostridium difficile* infection, graft-versus-host-disease of the gut, neutropenic enterocolitis/perforation, traumatic bowel perforation, bowel ischemia, bacterial peritonitis, ascending cholangitis, gastroparesis, pyelonephritis and/or kidney abscess, or other gastrointestinal surgery with complications. Twenty-one patients (84%) were immunosuppressed, including: 12 (48%) with underlying cancer and/or ongoing chemotherapy; 12 (48%) with diabetes mellitus (including 4 with an associated malignancy, 2 requiring hemodialysis for end-stage renal disease, 2 requiring immunosuppressive therapy for prior kidney-pancreas transplants, and 1 with hip osteomyelitis); and 1 patient requiring dialysis for end-stage renal disease due to hypertension. In-hospital mortality of patients with DNSE infection or colonization was high, occurring in 10 of 25 patients (40%).

Daptomycin exposure was confirmed in 15 of 25 (60%) patients prior to the isolation of DNSE. Of these, 10 of 15 (67%) had DNSE isolated during treatment with daptomycin after receiving an average of 13.9 days of therapy (range 3–40 days). The remaining 5 patients had recently received daptomycin with an average drug-free interval of 7.8 days (range 3–14 days) prior to DNSE isolation. In patients with prior daptomycin exposure, the total days of daptomycin therapy in the year prior to isolation of DNSE varied between 5–67 days, with a mean of 20.9 days and median of 16 days. Ten patients had no documented daptomycin exposure at our institution, and no evidence in their medical records that they received daptomycin prior to admission at UIHC. However, detailed records of care prior to UIHC admission were not always available and therefore daptomycin exposure could not be completely excluded in these cases.

Of patients with prior daptomycin exposure, 8 of 15 (53%) were female, compared to 6 of 10 (60%) of patients without prior daptomycin exposure (Table 2). Compared to patients without prior daptomycin exposure, patients with prior daptomycin exposure were less likely to harbor *E. faecalis* (0% vs. 33%; p = 0.019). In patients with DNSE and prior daptomycin exposure, there was a non-significant trend toward having a bloodstream isolate (47% vs. 20%; p = 0.174), a history of immunosuppression from any cause (93% vs. 70%; p = 0.119), and death (53% vs. 20%; p = 0.096).

**Table 2:** Characteristics of patients with respect to prior daptomycin exposure

	Prior Daptomycin Exposure	No Daptomycin Exposure	P value*
Number of Isolates	15 (60%)	10 (40%)	--
Age	52.5	51.8	--
Sex – no (%)			
-Female	8/15 (53%)	6/10 (60%)	0.742
Isolate			
-*E. faecium*	14 (93%)	4 (67%)	0.115
-*E. faecalis*	0 (0%)	2 (33%)	0.019
-Species Not Identified	1 (7%)	0 (0%)	--
-Isolate Not Available^a^	0	4	--
Source			
-Blood^b^	7 (47%)	2 (20%)	0.174
-Urine^b^	5 (33%)	7 (70%)	0.072
-Peritoneal Fluid	2 (13%)	1 (10%)	0.802
-Wound	2 (13%)	0 (0%)	0.229
-Colonization^c^	4 (27%)	4 (40%)	0.484
Past Medical History			
-Diabetes	6 (40%)	6 (60%)	0.327
-ESRD/Dialysis	2 (13%)	1 (10%)	0.802
-Cancer/Chemotherapy^d^	8 (53%)	4 (40%)	0.513
-History of Transplant	2 (13%)	0 (0%)	0.229
-CVD^e^	3 (20%)	4 (40%)	0.275
-Lung Disease^f^	2 (13%)	2 (20%)	0.656
GI or Intra-abdominal Process^g^	12 (80%)	7 (70%)	0.566
Immunosuppression^h^	14 (93%)	7 (70%)	0.119
Death^i^	8 (53%)	2 (20%)	0.096

Note:

* - All P values were calculated with the use of the chi-square test.

a – Four isolates were not available for species identification.

b – One patient with DNSE and prior daptomycin exposure had DNSE isolated from both blood and urine.

c – All colonizing isolates were obtained from urine of patients without symptoms and/or negative urinalysis.

d – includes all patients with acute lymphoblastic leukemia, acute myelogenous leukemia, myelodysplastic syndrome, bone marrow transplant, stem cell transplant, solid organ tumor, and those undergoing chemotherapy.

e – includes all patients with coronary artery disease, congestive heart failure, cerebro-vascular accident, peripheral vascular disease, and valvular heart disease.

f – includes all patients with chronic obstructive pulmonary disease and pulmonary hypertension.

g – includes all patients with *Clostridium difficile* infection, graft-versus-host-disease of the gut, neutropenic enterocolitis/perforation, traumatic bowel perforation, bowel ischemia, bacterial peritonitis, ascending cholangitis, gastroparesis, pyelonephritis and/or kidney abscess, or other gastrointestinal surgery with complications.

h – includes all patients with any cancer or undergoing chemotherapy, diabetes mellitus, ESRD/HD, organ transplant.

i – Hospital and/or thirty-day mortality from any cause.

**Figure 1:**
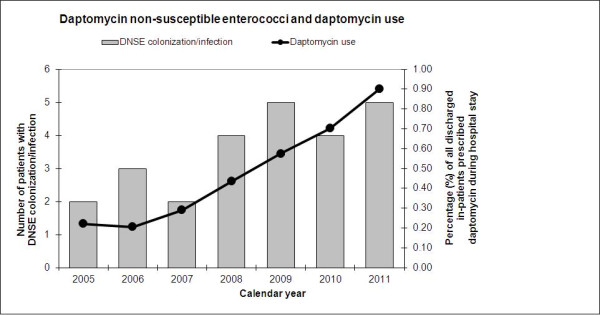
**Number of patients with colonization/infection due to daptomycin non-susceptible enterococci (solid bars; left vertical axis), and percentage of all discharged in-patients prescribed daptomycin during their hospital stay (solid line; right vertical axis) for each study year.** Information for 2011 based on information for the first 6 months of calendar year.

The number of patients with DNSE colonization or infection at UIHC increased from 2.33 cases per year for the time period 2005–2007 to 4.33 cases per year for the time period 2008–2010 (Figure 1). An additional five cases were identified in the first 6 months of 2011 alone – as many as had been noted over any 12-month period previously. Rates of daptomycin usage at UIHC increased during the study period as well, from approximately 0.2% of inpatients in 2005 receiving daptomycin to 0.9% in 2011 (Figure 1).

Twenty-one patients had enterococcal isolates available for species identification. Of these, eighteen patients (86%) had *E. faecium*, and two (10%) had *E. faecalis* (Table 3). Vancomycin resistance was common, occurring in DNSE isolates from 18 of 25 patients (72%), including 16 of 18 (89%) *E. faecium* isolates. Ampicillin resistance was detected in DNSE isolates from 18 of 25 patients (72%), and in 17 of 18 (94%) *E. faecium* isolates. Linezolid resistance was uncommon, occurring in only 1 DNSE isolate from 21 patients. Linezolid susceptibility was not performed for 4 patient’s isolates, as they were not available for further susceptibility testing.

**Table 3:** Antimicrobial resistance profile of enterococcal isolates to selected antimicrobial agents by species

Enterococcus species	Proportion of isolates resistant to selected antimicrobial agents			
	Daptomycin	Vancomycin	Ampicillin	Linezolid
	(%)	(%)	(%)	(%)
*E. faecium* (n = 18)	100	89	94	0
*E. faecalis* (n = 2)	100	50	0	0
Other (n = 5)^a^	100	20	20	5^b^

NOTE:

a – One Enterococcus isolate was identified as non-faecium / non-faecalis, and four were not available for species identification or linezolid susceptibility testing.

b – Of the 21 patient isolates available for susceptibility testing; only the non-faecium / non-faecalis Enterococcus isolate was resistant to linezolid.

PFGE revealed significant genetic heterogeneity, with 16 PFGE types and 24 subtypes represented among the 29 patient isolates available for typing (results not shown). Multiple isolates from the same patient were always of the same type or subtype. There were only four instances in which more than one patient shared the same PFGE type with the most common PFGE type shared among 4 patients. In only one instance did two patients share the same PFGE subtype.

## Discussion

In the past 20 years, there have been increases in both the frequency of enterococcal infections and in rates of enterococcal drug resistance [12]. VRE is now the third most common cause of nosocomial bloodstream infection [13], and compared to vancomycin-susceptible enterococci (VSE), is associated with increased healthcare costs, morbidity, and mortality [14]. Enterococci are of particular concern due to their ability to spread drug resistance to other bacteria via mobile genetic elements, resulting in beta-lactam, aminoglycoside, and glycopeptide resistance.

Vancomycin resistance in both *Enterococcus* species and *S. aureus* is well described, mediated by van-type plasmids from VRE [15]. In *S. aureus*, daptomycin resistance is possibly mediated by several mechanisms, including cell wall thickening and charge alterations [16,17]. The exact mechanism of daptomycin resistance in enterococci is not entirely known, but may be related to alterations in cell membrane charge, thickness, and permeability via mutations to cell membrane cardiolipin synthetase and/or other proteins involved in regulating phospholipid metabolism or the stress response to antimicrobial agents [18,19]. Daptomycin exposure is likely to be essential to the development of resistance in enterococci as suggested by the findings in the aforementioned studies and our finding of a high rate (60%) of confirmed prior daptomycin exposure in patients harboring a DNSE isolate as well as the genetic heterogeneity noted on PFGE.

Little is known regarding risk factors or causes for the development of DNSE. Prior daptomycin exposure has been described in case reports of DNSE bacteremia; however cases describing resistance developing de novo have been reported [5-9]. In the largest case series to date of DNSE bacteremia, performed at Memorial Sloan-Kettering Cancer Center during a 3 year period (2007–2009), the authors found prior daptomycin exposure to be an uncommon event with only 2 of 18 (11%) patients having had documented daptomycin exposure prior to the development of DNSE bacteremia [7]. These findings are in contrast to our observation at UIHC, where during a similar time period (2005–2011), the majority of patients with DNSE colonization or infection had confirmed daptomycin exposure prior to the isolation of a non-susceptible isolate (any clinical sample; 15 of 25 or 60%, blood stream 7 of 9 or 78%). This may be an under-estimate however, as the rate of confirmed daptomycin exposure in the era of electronic order entry (February 2009 to present) at UIHC was substantially higher than before its institution (86% vs. 27%). A recent case series and case control study comparing DNSE in patients either with or without prior daptomycin exposure showed results similar to ours, with prior daptomycin exposure in 59%, immunosuppression in 78%, an average age of 58.9 years, *E. faecium* in 78%, and death in 44% [8,9].

Since daptomycin was introduced for clinical use in 2003, significant increases in rates of resistance or MIC creep have not been noted in the United States with an overall prevalence of DNSE estimated at < 1% [4,20,21]. However in other areas, in particular Asia and parts of Europe, resistance rates may be significantly higher than in the US [4]. Over the past 6 years at our institution, we have noticed an increase in the absolute number of patients colonized or infected with DNSE, which has taken place in the setting of increasing rates of daptomycin usage during the same period (Figure 1).

In the US, approximately 90% of DNSE isolates are also resistant to vancomycin [20,21]. And like VRE, daptomycin non-susceptible isolates are more often *E. faecium* than *E. faecalis* (88% vs. 9%) [4]. At our institution a large majority (99%) of clinical enterococcal isolates remain daptomycin susceptible, and the observed species identification and susceptibility of the daptomycin non-susceptible isolates observed in our study align with that reported nationally (Table 3) [4,20].

In addition to prior daptomycin exposure, several findings in patients with DNSE colonization or infection at our institution are worth noting and may provide clues to potential factors associated with the development of DNSE. The majority of patients were immunosuppressed (21 of 25; 84%) or had a concomitant gastrointestinal inflammatory process (19 of 25; 76%). Also, a genitourinary process was noted in 3 of 25 (12%) patients. These results are not surprising given that enterococci commonly colonize the gastrointestinal and genitourinary tracts, and that the majority of these patients had risk factors for the development of gastrointestinal or genitourinary complications or infections.

Molecular typing revealed that daptomycin non-susceptibility emerged among diverse strains of enterococci, with patient-to-patient transmission of DNSE occurring less often. This suggests that cases of DNSE were likely the result of mutations in patient’s own flora under antimicrobial pressure, as opposed to patient-to-patient transfer of an outbreak or common strain. Finally, in-hospital mortality of patients with DNSE infection or colonization was high, at 40%. We are not aware of outcome studies related to the presence of daptomycin non-susceptibility versus daptomycin susceptibility in enterococcal colonization or infection.

## Conclusion

To our knowledge, this is the largest study to date to evaluate potential factors associated with the development of DNSE colonization and/or infection and to compare the characteristics of patients with prior daptomycin exposure to those without prior daptomycin exposure. We found that prior daptomycin exposure, immunosuppression, or a concomitant gastrointestinal or intra-abdominal process, preceded the development of DNSE. During the study period, we noticed an increase in the number of cases of DNSE yearly that corresponded to increasing annual rates of daptomycin usage at our institution. Molecular typing revealed a diverse population of isolates suggesting the development of resistance under antimicrobial pressure. Also, a high percentage of patients with DNSE colonization or infection died during their hospitalization. Limitations of this study include its retrospective nature and small number of patients. Future case–control or prospective studies comparing patients with and without DNSE and/or prior daptomycin exposure would be helpful in better identifying factors associated with DNSE infection and/or colonization.

## Competing interest

Dr. Diekema has received research funding from Merck, Pfizer, Cerexa, Innovative Biosensors, and bioMérieux. All other authors have no disclosures or conflict of interest.

## Authors’ contribution

All authors have read and approve the submission of the manuscript. The manuscript has not been published elsewhere and that it is not currently under consideration for publication by another journal.

## References

[B1] Gold HS Vancomycin-resistant enterococci: mechanisms and clinical observations Clin Infect Dis 2001 33 2 210 219 10.1086/321815 11418881

[B2] Centers for Disease Control and Prevention Staphylococcus aureus resistant to vancomycin—United States, 2002 MMWR Morb Mortal Wkly Rep 2002 51 26 565 567 12139181

[B3] Linden PK Optimizing therapy for Vancomycin Resistant Enterococci (VRE) Sem Resp Crit Care Med 2007 28 6 632 645 10.1055/s-2007-996410 18095227

[B4] Kelesidis T Humphries R Uslan DZ Pegues DA Daptomycin nonsusceptible enterococci: an emerging challenge for clinicians Clin Infect Dis 2011 52 2 228 234 10.1093/cid/ciq113 21288849 PMC8483151

[B5] Lesho EP Wortmann GW Craft D Moran KA De Novo daptomycin nonsusceptibility in a clinical isolate J Clin Microbiol 2006 44 2 673 10.1128/JCM.44.2.673.2006 16455945 PMC1392681

[B6] Fraher MH Corcoran GD Creagh S Feeney E Daptomycin non-susceptible enterococci faecium in a patient with no prior exposure to daptomycin J Hosp Infect 2007 65 4 376 378 10.1016/j.jhin.2007.01.002 17316896

[B7] Kamboj M Cohen N Gilhuley K Babady NE Seo SK Sepkowitz KA Emergence of daptomycin-resistant VRE: experience of a single institution Infect Control Hosp Epidemiol 2011 32 4 391 394 10.1086/659152 21460492 PMC3676937

[B8] Kelesidis T Humphries R Uslan DZ Pegues D De-novo daptomycin nonsusceptible Enterococcal infections Emerg Infect Dis 2012 18 4 674 676 10.3201/eid1804.110932 22469288 PMC3309676

[B9] Kelesidis T Chow ALP Humphries R Uslan DZ Pegues D Case–control study comparing de novo and daptomycin-exposed daptomycin-nonsusceptible Enterococcus infections Antimicrob Agents Chemother 2012 56 4 2150 2152 10.1128/AAC.05918-11 22252808 PMC3318388

[B10] Clinical and Laboratory Standards Institute (CLSI) Performance Standards for Antimicrobial Susceptibility testing – Twentieth Information Supplement 2010 CLSI, Wayne, PA CLSI document M100-S20

[B11] Tenover FC Arbeit RD Goering RV Mickelsen PA Murray BE Persing DH Swaminathan B Interpreting chromosomal DNA restriction patterns produced by pulsed-field gel electrophoresis: criteria for bacterial strain typing J Clin Microbiol 1995 33 9 2233 2239 7494007 10.1128/jcm.33.9.2233-2239.1995 PMC228385

[B12] Centers for Disease Control and Prevention National Nosocomial Infections Surveillance (NNIS) system report, data summary from January 1992 through June 2004, issued October 2004 Am J Infect Control 2004 32 470 485 10.1016/j.ajic.2004.10.001 15573054

[B13] Wisplinghoff H Bischoff T Tallent SM Seiferd H Wenzel RP Edmond MB Nosocomial bloodstream infections in US hospitals: analysis of 24,179 cases from a prospective nationwide surveillance study Clin Infect Dis 2004 39 309 317 10.1086/421946 15306996

[B14] Carmeli Y Eliopoulos G Mozaffari E Samore M Health and economic outcomes of vancomycin-resistant enterococci Arch Intern Med 2002 162 2223 2238 10.1001/archinte.162.19.2223 12390066

[B15] Arthur M Reynolds PE Depardieu F Evers S Dutka-Malen S Quintiliani R Courvalin P Mechanisms of glycopeptide resistance in enterococci J Infect 1996 32 11 16 10.1016/S0163-4453(96)80003-X 8852545

[B16] Mishra NN Yang SJ Sawa A Rubio A Nast CC Yeaman MR Bayer AS Analysis of cell membrane characteristics of in vitro-selected daptomycin-resistant strains of methicillin-resistant staphylococcus aureus Antimicrob Agents Chemother 2009 53 6 2312 2318 10.1128/AAC.01682-08 19332678 PMC2687258

[B17] Jones T Yeaman MR Sakoulas G Yang SJ Proctor RA Sahl HG Schrenzel J Xiong YQ Bayer AS Failures in clinical treatment of Staphylococcus aureus infection with daptomycin are associated with alterations in surface charge, membrane phospholipid asymmetry, and drug binding Antimicrob Agents Chemother 2008 52 269 278 10.1128/AAC.00719-07 17954690 PMC2223911

[B18] Palmer KL Daniel A Hardy C Silverman J Gilmore MS Genetic basis for daptomycin resistance in enterococci Antimicrob Agents Chemother 2011 55 7 3345 3356 10.1128/AAC.00207-11 21502617 PMC3122436

[B19] Arias CA Panesso D McGrath DM Qin X Mojica MF Miller C Diaz L Tran TT Rincon S Barbu EM Reyes J Roh JH Lobos E Sodergren E Pasqualini R Arap W Quinn JP Shamoo Y Murray BE Weinstock GM Genetic basis for in vivo daptomycin resistance in enterococci N Engl J Med 2011 365 10 892 900 10.1056/NEJMoa1011138 21899450 PMC3205971

[B20] Sader HS Jones RN Antimicrobial susceptibility of gram-positive bacteria isolated from US medical centers: results of the Daptomycin Surveillance Program (2007–2008) Diagn Microbiol Infect Dis 2009 65 158 162 10.1016/j.diagmicrobio.2009.06.016 19748426

[B21] Sader HS Moet GJ Farrell DJ Jones RN Antimicrobial susceptibility of daptomycin and comparator agents tested against methicillin-resistant Staphylococcus aureus and vancomycin-resistant enterococci: trend analysis of a 6-year period in US medical centers (2005–2010) Diagn Microbiol Infect Dis 2011 70 412 416 10.1016/j.diagmicrobio.2011.02.008 21546202

